# Comparative Mutational Profiling of Hematopoietic Progenitor Cells and Circulating Endothelial Cells (CECs) in Patients with Primary Myelofibrosis

**DOI:** 10.3390/cells10102764

**Published:** 2021-10-15

**Authors:** Mirko Farina, Simona Bernardi, Nicola Polverelli, Mariella D’Adda, Michele Malagola, Katia Bosio, Federica Re, Camillo Almici, Andrew Dunbar, Ross L. Levine, Domenico Russo

**Affiliations:** 1 Unit of Blood Diseases and Bone Marrow Transplantation, Cell Therapies and Hematology Research Program, Department of Clinical and Experimental Sciences, University of Brescia, ASST Spedali Civili di Brescia, P.le Spedali Civili, 1, 25123 Brescia, Italy; simona.bernardi@unibs.it (S.B.); nicola.polverelli@unibs.it (N.P.); michele.malagola@unibs.it (M.M.); k.bosio001@unibs.it (K.B.); federicare91@gmail.com (F.R.); domenico.russo@unibs.it (D.R.); 2 CREA Laboratory (Centro di Ricerca Emato-Oncologica AIL), ASST Spedali Civili di Brescia, 25123 Brescia, Italy; 3 Hematology Unit, ASST Spedali Civili di Brescia, 25123 Brescia, Italy; mariella.dadda@asst-spedalicivili.it; 4 Laboratory for Stem Cells Manipulation and Cryopreservation, Department of Transfusion Medicine, ASST Spedali Civili di Brescia, 25123 Brescia, Italy; camillo.almici@asst-spedalicivili.it; 5 Center for Hematologic Malignancies, Memorial Sloan Kettering Cancer Center, New York, NY 10065, USA; dunbara@mskcc.org (A.D.); leviner@mskcc.org (R.L.L.)

**Keywords:** hematopoiesis-stem and primitive progenitor cells, circulating endothelial cells, myelofibrosis, molecular genetics, vascular biology-endothelial cells

## Abstract

A role of endothelial cells (ECs) in Primary Myelofibrosis (PMF) was supposed since *JAK2* mutation was found in endothelial precursor cells (EPCs) and in ECs captured by laser microdissection. By Cell Search method, the circulating endothelial cells (CECs) from 14 PMF patients and 5 healthy controls have been isolated and compared by NGS with CD34+Hematopoietic stem and progenitors cells (HSPCs) for panel of 54 myeloid-associated mutations. PMF patients had higher levels of CECs. No mutation was found in HSPCs and CECs from controls, while CECs from PMF patients presented several somatic mutations. 72% of evaluable patients shared at least one mutation between HSPCs and CECs. 2 patients shared the *JAK2* mutation, together with *ABL1*, *IDH1*, *TET2* and *ASXL1*, *KMT2A*, respectively. 6 out of 8 shared only NON MPN-driver mutations: *TET2* and *NOTCH1* in one case; individual paired mutations in *TP53*, *KIT*, *SRSF2*, *NOTCH1* and *WT1*, in the other cases. In conclusion, 70% of PMF patients shared at least one mutation between HSPCs and CECs. These latter harbored several myeloid-associated mutations, besides *JAK2V617F* mutation. Our results support a primary involvement of EC in PMF and provide a new methodological approach for further studies exploring the role of the “neoplastic” vascular niche.

## 1. Introduction

Primary Myelofibrosis (PMF) is a myeloproliferative neoplasm (MPN) characterized by clonal myeloproliferation, deregulated cytokine production and bone marrow (BM) fibrosis. Splenomegaly, constitutional symptoms, progressive anemia and/or thrombocytopenia dominate the clinical picture of the disease [1,2].

While the pathogenesis is not yet completely elucidated, the biological hallmark of PMF consists of an aberrant activation of JAK-STAT pathway derived from the mutation in the MPN driver genes, *JAK2 V617F* (50–60%) [3,4], Calreticulin (*CALR*) (20–25%) [4,5] and *MPL* (5%) [4,6]. Furthermore, about 5 to 10% of PMF patients do not carry any MPN driver mutations and are defined as “triple negative” [5].

Recently, thanks to the use of Next Generation Sequencing (NGS) technologies, somatic mutations have been found in almost 90% of PMF patients. Some of them, such as *ASXL1, DMT3A, EZH2, IDH1/IDH2* and *SRSF2*, are known to be associated with a worsened clinical course and higher risk of leukemic transformation and thus are defined as “high molecular risk mutations” [3,7].

Characteristically, PMF patients also present with a higher rate of vascular complications [8,9,10] and increased BM and spleen vascularity [11]. Considering these features and the physiological role of JAK-STAT pathway in preserving the endothelial-vascular homeostasis [12], it has been supposed that endothelial cells (ECs) have a role in the pathogenesis of PMF and other MPNs [13,14]. To explore this hypothesis, some studies have investigated the presence of *JAK2 V617F* mutation in MPN patients’ ECs and its role as predictor of thrombosis [13,14,15]. Unfortunately, the results of these studies are discordant. At first, some authors tried to detect the *JAK2* mutation in endothelial progenitors cells (EPCs) derived from MPN patients and cultured in vitro. The *JAK2* mutation was found in the so-called “colony forming unit-endothelial cells” (CFU-ECs) [16,17,18], but these cells are now no longer considered as true EPCs. Conversely, “Endothelial Colony Forming Cells” (ECFCs) were shown to form ECs colonies in vitro and to generate new vessels in vivo. For these reasons, their role as true EPC [19] seem very likely. ECFCs are increased in PMF patients [20], but it is still debated whether they can independently harbor the *JAK2 V617F* mutation or not [15]. While several authors repeatedly documented that ECFCs do not carry the *JAK2* mutation [21,22], Teofili found that ECFCs from a subset of MPN patients with a previous history of thrombosis may carry this mutation [23]. In addition, the *JAK2* mutation was detected also in BM-derived ECFCs [24]. Confirming the endothelium involvement in MPNs, the *JAK2* mutation was also detected in the mature ECs captured by laser microdissection from spleen and hepatic vessels in MPN patients [21,25]. However, due to ethical and practical reasons searching for mutated ECs through the technique of microdissection in organs is strongly limited in vivo and therefore does not allow for the systematic study of ECs in patients.

Regardless, the results of these studies, the high incidence of vascular events in MPNs, and the role of BM and spleen in neoangiogenesis strongly suggests that ECs may be involved in the development and progression of PMF. However, some open questions remain. In particular, it’s still not clear if ECs may be primary involved in PMF development or not. Moreover, it’s argued how ECs might acquire the *JAK2* mutation. For this latter aspect, an intriguing hypothesis is that ECs and hematopoietic stem and progenitors cells (HSPCs) may share a common progenitor cell.

In the present study (MyCEC0617), we detect and evaluate circulating endothelial cells (CECs) isolated from PMF patients and healthy controls using the Cell Search method. CECs are mature ECs detached from endothelium following ECs turnover or vascular injury [26,27] and are increased in MPN patients [28]. Moreover, for the first time, we have comparatively evaluated, both in CECs and CD34 + HSPCs, a panel of 54 myeloid-associated somatic mutations beyond the MPN drivers *JAK2, MPL* and *CALR.*

## 2. Patients and Methods

### 2.1. Patients and Healthy Controls

Between July 2018 and July 2020, we prospectively evaluated 14 PMF patients and 5 healthy subjects, as controls. The MyCEC0617 study was approved by the local Ethical Committee and in accordance with the Helsinki II Declaration. All subjects gave written informed consent. Only patients and healthy controls over 18 years old and with a performance status greater or equal to 2 (ECOG score) were eligible for the study. In addition, patients must be diagnosed with PMF and not being previously treated with JAK-STAT inhibitors (treatment with Hydroxyurea was permitted). These inclusion criteria were thought to avoid any possible bias or confounding factors deriving by the use of JAK-STAT inhibitors or by a previous history of Polycythemia Vera or Essential thrombocythemia.

The disease status at the time of samples collection was evaluated using the Dynamic International Prognostic Scoring System (DIPSS) [29]. 

### 2.2. Study Plan

**Figure 1:**
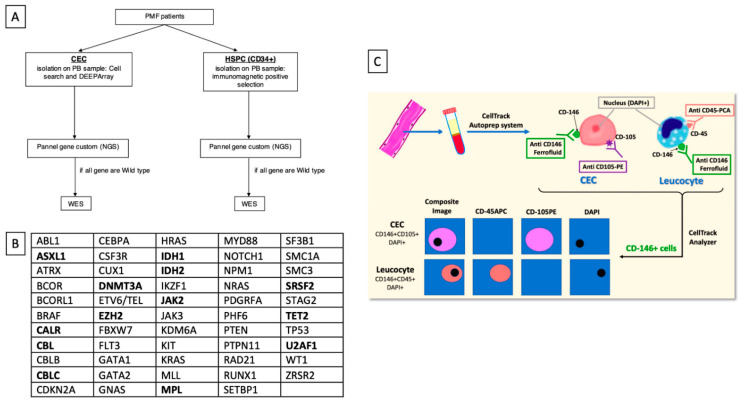
Study plan and CellSearch technologies. The study plan (**A**) and the 54-myeloid associated genes panel (**B**) used to investigate DNA from HSPCs and CECs. In bold the genes that are more closely related to myelofibrosis [3,4,30,31]. CECs were identify using the CellSearch system (**C**). Tubes containing 10 mL of peripheral blood are centrifuged to separate blood into plasma, buffy coat and red blood cell layer. The blood tube is then placed into the CellTrack Autoprep system where blood cells are incubated with antibodies against CD146, CD105, CD45 and are stained with DAPI. In this step, CD146-positive CECs are labeled with anti-CD105-PE antibodies while leukocytes are labeled with anti-CD45-APC antibodies. The labeled cells are then analyzed and enumerated in CellTracks Analyzer. CECs are identified as CD105-positive/DAPI-positive/CD45-negative cells while leukocytes are identified as CD45-positive/DAPI-positive/CD105-negative cells.

The MyCEC0617 study plan is summarized in Figure 1A. Briefly, in PMF patients or healthy controls, two samples of peripheral blood (PB) (10 mL each) were collected: one for CECs detection, and one for HSPCs selection. DNA from both CECs and HSPCs was then investigated using a 54-gene custom panel focused on genes mutated in PMF [3,4,30,31] (Figure 1B). If no mutations were detected, then Whole Exome Sequencing (WES) was performed only for PMF patients. 

### 2.3. CD34 + HSPC Detection and Selection

For CD34 + HSPC detection, 10 mL of PB was collected in EDTA (Ethylenediaminetetraacetic acid) tubes and examined within 6 h. HSPCs were selected using CD34+ immunomagnetic bead-column separation (magnetic-activated cell sorting (MACS) CD34 MicroBead Kit by Miltenyi biotech, 51429 Bergisch Gladbach, Germany). Specifically, the mononuclear cells (MNCs) layer obtained after Ficoll centrifugation (Lymphosepar I; IBL, Gunma, Japan) were magnetically labeled with CD34 MicroBeads [32]. Then, the cell suspension was loaded into a MACS Column, which was placed in the magnetic field of a MACS Separator. The unlabeled cells ran through while the magnetically labeled cells were retained on the MACS Column. The retained material was then washed with buffer to remove unlabeled material. After removing the column from the magnetic field, the magnetically retained CD34+ cells were eluted as the positively selected cell fraction and counted using the Bürker-Turk chamber [33].

### 2.4. CellSearch CECs Identification and Collection

For CECs analysis, 10 mL of PB were collected in dedicated tubes containing a cell preservative (CellSave Preservative Tubes; Veridex LLC, Raritan, NJ, USA). All samples were stored at room temperature, shipped via overnight express courier to a referral Laboratory (Menarini Silicon Biosystems Laboratory, Bologna, Italy), and processed within 96 h as previously described [34]. CellSearch system is an immunomagnetic selection-based approach incorporating ferrofluid nanoparticles (anti-CD146) and fluorophore-labelled antibodies (anti CD105, anti CD45 and DAPI) (Figure 1C). The CellSearch system consists of two instruments: the CellTrack Autoprep and the Analyzer. Briefly, tubes containing blood are centrifuged to separate blood into plasma, buffy coat and red blood cell layer. The blood tube was then placed into the CellTrack Autoprep system where blood cells were incubated with a ferrofluid against CD146 (immunomagnetic selection). CD146, also known as the melanoma cell adhesion molecule (MCAM), is a cell adhesion molecule currently used as a marker for endothelial cell lineage. Then, CD146 positive cells were stained with labelled antibodies against CD105 (an endoglin protein expressed by activated ECs, monocytes, stromal cells and pre-B cells) and CD45 (expressed by leukocytes), and with the nuclear stain 4,6-diamidino-2-phenylindole (DAPI). Thereafter, the labeled cells were analyzed and enumerated in the CellTracks Analyzer, a four-colour semi-automated fluorescent microscope. CECs were identified as CD105-positive/DAPI-positive/CD45-negative cells, while leukocytes were identified as CD45-positive/DAPI-positive/CD105-negative cells (more details in Appendix A). 

Subsequently, putative CECs were sorted using the DEPArray system (Di-Electro-Phoretic Array system; by Menarini Silicon Biosystems, Bologna, Italy) [35], a semi-automated device that allows to isolate rare cells from mixed-cell populations at the single-cell level [36], combining di-electrophoresis technology and high-quality image-based cell selection. The DEPArray system is composed of three elements: a benchtop instrument, a disposable microfluidic cartridge and a proprietary software, the CellBrowser. The working principle of the DEPArray is the Dielectrophoresis (DEP), an electrokinetic principle based on the ability of a non-uniform electric field to exert forces on neutral, polarizable particles, such as cells, which are suspended in a liquid. The core of the DEPArray technology is the microsystem cartridge, which is a single-use device integrating a microelectronic silicon chip (over 300,000 micro-electrodes), microfluidic chambers and valves. Briefly, fluorescently labeled cells can be visualized and isolated by means of a chip consisting of various microelectrodes creating electric cages in which individual cells are trapped. Alternatively activating and deactivating the microelectrodes on the chip results in moving the caged cells to a position in the chip that allows the recovery of these cells in a medium suitable for downstream analysis (for more details, please see Appendix B). Following the manufacturer’s instructions and the standard procedure, the final volume of CECs collection was 4 mL of PB [35].

### 2.5. NGS Analysis

DNA extracted from isolated CECs and HSPCs was amplified in order to obtain a quantity suitable for NGS analysis. The amplification was performed using Reply-G Single Cell WGA kit (Qiagen, Germantown, Germany) following the manufacturer’s instructions. Sequencing data was then assessed with the MiSeq Illumina NGS platform using a custom gene panel including 54-genes know to be recurrently mutated in PMF (Figure 1B). Our approach was based on the gene target capture sequencing. Specific probes (NimbleGen by Roche, Madison, WI, USA) have been used in order to hybridize all exons of the above-mentioned genes (141 kb), as previously described [37]. The captured sequences of CEC and HSPC DNA from 4 patients were thus pooled (8 samples per pool) [38] and sequenced following manufacturer’s instructions by MiSeq Illumina NGS platform using 2 × 150 sequencing (V2 kit, TruSeq, San Diego, CA, USA). One sequencing run was required in order to sequence 8 samples with a coverage about 3200× [39]. The .vcf files were analyzed using the free bioinformatics tool wAnnovar (Wang Genomics Lab 2010–2020) [40]. Integrative Genomics Viewer (IGV) [41] was used to analyze the presence of big deletions in the sequenced loci. The cutoffs to confirm the presence of the mutations were the identification of mutant alleles in 30 and 50 reads for HSPC and CEC, respectively, both in forward and reverse strand (see Appendix C).

### 2.6. Statistical Analysis

Standard descriptive statistics were used to summarize the patient samples. Continuous data were expressed as median (range). Categorical variables were compared using the chi-square or Fisher’s exact test. Mann-Whitney U test was used in univariate analysis for comparison of continuous variables. The clinical and laboratory parameters, as well as comorbid conditions (for more details please see Supplementary Materials) and PMF treatments, were analyzed as possible factors related to the presence of molecular mutations on CECs and HSPCs and to the detection of shared mutations between the two subpopulations. Overall survival was calculated from the date of sample collections to the last follow up or death, using the Kaplan-Meier method; the log-rank test was used to evaluate differences among subgroups. The cumulative incidence of acute myeloid leukemia (AML) progression in patients who shared somatic mutations and those who did not was performed with mortality as competing risk. Comparisons between cumulative incidences were performed using the Gray test. All reported P values are two-sided, and P values of less than 0.05 were considered to indicate statistical significance. Statistical analyses were performed with EZR software (v1.40) [42]. For original data, please contact mirkfar@gmail.com. 

## 3. Results

### 3.1. Patients and Healthy Controls Characteristics

The main characteristics of patients and healthy controls are reported in Table 1. All patients were diagnosed with PMF. Their median age was 71.5 years, male sex was predominant (64%) and the median time from diagnosis to sample collection was 20.5 months. Nine of the 14 patients were *JAK2* mutated, 2 were CALR mutated and 2 MPL W515L. One patient was triple-negative. The mutational status was evaluated by conventional PCR followed by Sanger Sequencing according to the routine MPN patients’ management. Overall, 11 of the 14 patients had splenomegaly, while two patients experienced thrombosis before being diagnosed (one portal vein thrombosis, and one central retinal artery occlusion). Most of the patients presented White blood cells (WBC) and platelets (PLT) count in normal range at the time of sample collections (2 patient presented hyperleukocytosis; 3 had high platelets count; 2 patients had thrombocytopenia), while median hemoglobin level was 10.7 g/dL. Most of the patients (*n* = 7) had an Intermediate-1 DIPSS score, 5 were intermediate-2 and 2 high-risk DIPSS score. 71% of patients didn’t receive any treatment at or prior the time of sample collection, while four patients were receiving hydroxyurea as cytoreductive treatment. Two of them had been receiving the drug from the diagnosis, for a total of 2 months each; while the other two had been receiving treatment for 12 and 14 months, respectively. (For more details on patients and healthy controls characteristics please see Table 1 and Supplementary Table S1).

The 5 healthy controls had no known illness or history of malignant disease or thrombosis. Their clinical features and peripheral blood counts are reported in Table 1.

The median follow-up from samples collection was 24 months (3–29) and it was not different between patients who shared mutations between CECs and HSPCs [24.5 months (10.5–25.2)] and who did not [29 months (24–29)] (p: 0.16).

### 3.2. CEC and HSPCs Enumeration and Collection

**Figure 2:**
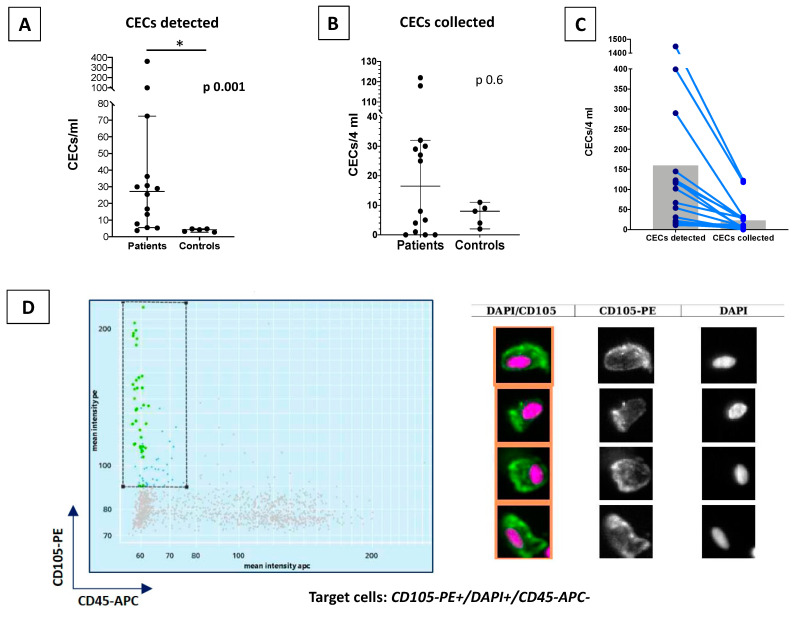
CellSearch detection of CECs and DEPArray imaging. (**A**) The CECs detected per mL in PMF patients and healthy controls. PMF patients presented a significative higher level of CECs (*p* = 0.001). (**B**) The CECs collected per mL in PMF patients and healthy controls. (**C**) The CECs quantitative difference comparing the CECs detection and collected levels. (**D**) DEPArray imagines comparision. On the left, the DEPArray scatter plot, which is based on mean fluorescence intensity and with the gate for CD105-PE positive (Y axis) and CD45-APC negative (X axis) cells. On the right, the original Cell Search images. In the first column the cells selected as CECs, which presented in purple the nuclear stain DAPI, while in green the CD105 staining. In the second column the selection of CD105-PE staining, while the third shown the DAPI staining. CECs were defined as CD105PE+/DAPI+/CD45APC-. The CECs median comparison was made using the Mann-Whitney test. * *p* < 0.05.

By CellSearch system, CECs were successfully detected in all samples (14 PMF patients and 5 controls) (Table 2, Supplementary Table S2). PMF patients showed significant higher levels of CECs (25.5/mL; range: 3.75–362/mL) compared with healthy controls (4.25/mL; range: 2.75–4.75) [*p* = 0.001; Table 2; Figure 2A]. A previous history of thrombosis was associated with a higher, but not significant, level of CECs (*p* = 0.30) (Table 2). The number of CECs was not related with any of the other variables analyzed (Table 2). After isolation by CellSearch technology, the CECs were managed by the DEPArray system for their sorting (Figure 2). CECs recoveries were performed successfully in 11 out of 14 patients and in all healthy controls (Supplementary Table S2). 

In particular, a median of 8 CECs in 4 mL of PB were collected in healthy controls (range: 2–11), while a median of 26 CECs/4 mL of PB were isolated in PMF patients (range: 1–122) (Figure 2B,C; Supplementary Table S2). 

A median of 6.15 × 10^4^ HSPCs/mL were collected in PMF patients (0.7–12.7), while 3.2 × 10^4^ HSPCs/mL were collected in healthy controls (*p* = 0.15) (Supplementary Table S2). 

### 3.3. Comparative NGS Analysis on PMF Patients’ HSPCs and CECs 

Of note, no mutation was found in HSPCs and CECs from healthy controls, in whom known polymorphisms in both the cells subpopulations were only observed. On the contrary, a number of somatic mutations in both HSPCs and CECs were assessed in PMF patients. 

**Figure 3:**
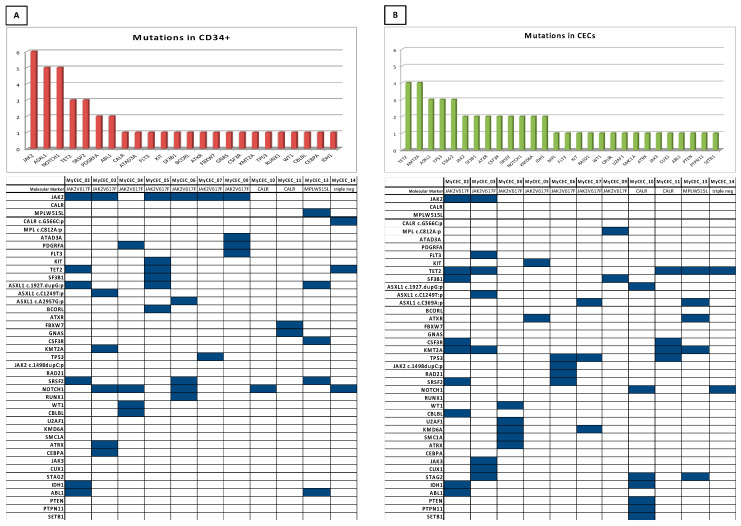
Molecular alterations on CD34^+^-HSPCs and CECs. Mutated gene frequency and Molecular alterations discovered on CD34+-HSPCs (**A**) and CECs (**B**). On the top the frequency of mutated genes, while on the bottom the table with all the mutated genes in each patient.

The previously-identified MPN driver mutations were confirmed by NGS on PMF patients’ HSPCs in all cases, except for one out of the six *JAK2*-mutated patients and for the two *CALR*-mutated patients, who presented *CALR* mutation under the detection limit (Figure 3A, Supplementary Table S2). 

In HSPCs, 24 of the 54 genes analyzed were mutated, with a median of 4 mutations (1–6) per cell and a variant allele frequency (VAF) of 5%, at least (Supplementary Table S2). The most frequent mutated gene was *JAK2* (6 patients), followed by *ASXL1*, *NOTCH1* (5 patients) and by *TET2* and *SRSF2* (3 patients). Overall, five patients harbored high molecular risk mutations (*ASXL1, IDH1/2, SFRSF2, EZH1*) [3] in HSPCs (Figure 3A).

Interestingly, a median of 4 (2–9) mutations/patient was detected in CECs isolated from PMF patients (Figure 3B, Supplementary Table S2). Overall, 28 different genes were mutated in CECs, with a VAF of 5%, at least. The *JAK2 V617F* mutation was found in 2 of the 6 *JAK2*^+^ patients (33.3%), while neither CALR nor MPL driver mutations were found in CECs. *TET2, KMT2A, ASXL1, TP53* and *STAG2* were the genes more frequently mutated in CECs (Figure 3B). In particular, *TET2* and *KMT2A* were altered in 4 patients, while *ASXL1, TP53* and *STAG2* in 3 patients. Overall, no relationships were found between the clinical characteristics and the number or type of genes mutated in the CECs.

**Figure 4:**
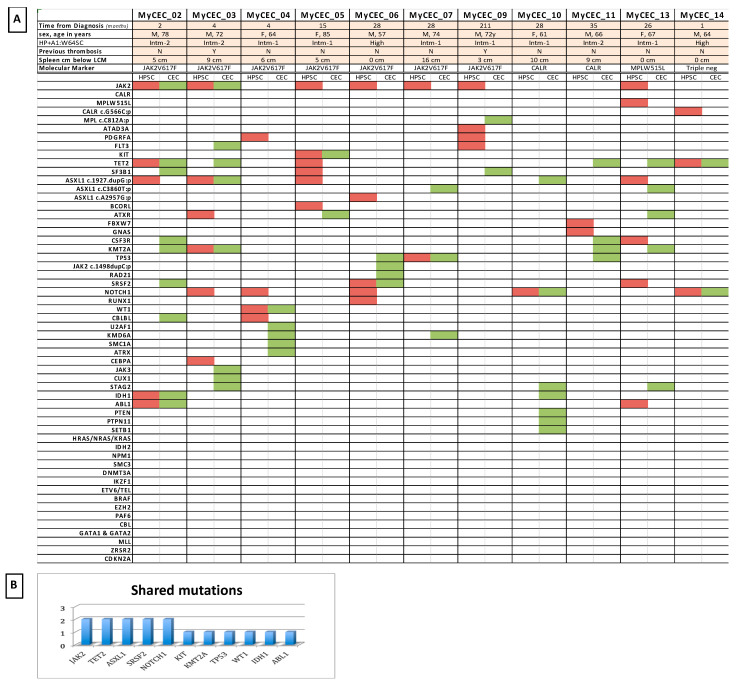
Comparative Somatic profiling of CD34^+^-HSPCs and CECs in PMF patients. (**A**) Molecular profiles of both CEC and HSPC in patients with PMF. The molecular lesions found in the HSPC are in red, while in Green the ones discovered in the CEC. At the top of the table the clinical characteristics of patients, who successfully recovered CEC. (**B**) Mutated genes shared between HSPCs and CECs.

When comparing mutational profiles of HSPCs and CECs in PMF patients, 8 of 11 patients (72.7%) shared at least one mutation in the two subpopulations (Figure 4). Two of the six *JAK2^+^* patients shared the MPN driver mutation between HSPCs and CECs and they were showed also the highest number of shared mutations: *ABL1, IDH1* and *TET2* in one case*,* and in *ASXL1* and *KMT2A* in the other case. No other shared MPN driver mutations were found in CECs and HSPCs. Six of the 8 patients shared only NON MPN-driver somatic mutations between the two cells’ subpopulations: *TET2* and *NOTCH1* in one case, and individual paired mutations in *TP53, KIT, SRSF2, NOTCH1* and *WT1*, in the other 5 patients. 

Considering the polymorphic alleles, in the loci analyzed we didn’t find loss of the heterozygosity (LOH) in HSPCs in any PMF patients, while the CECs from 3 out of 11 patients presented LOH in different loci (*GATA2 C15G; P5P*; *PDGFRA C2472T; V824V*; and *JAK2 G2490A; L830L* on MyCEC04, MyCEC09 and MyCEC06 patients, respectively).

**Figure 5:**
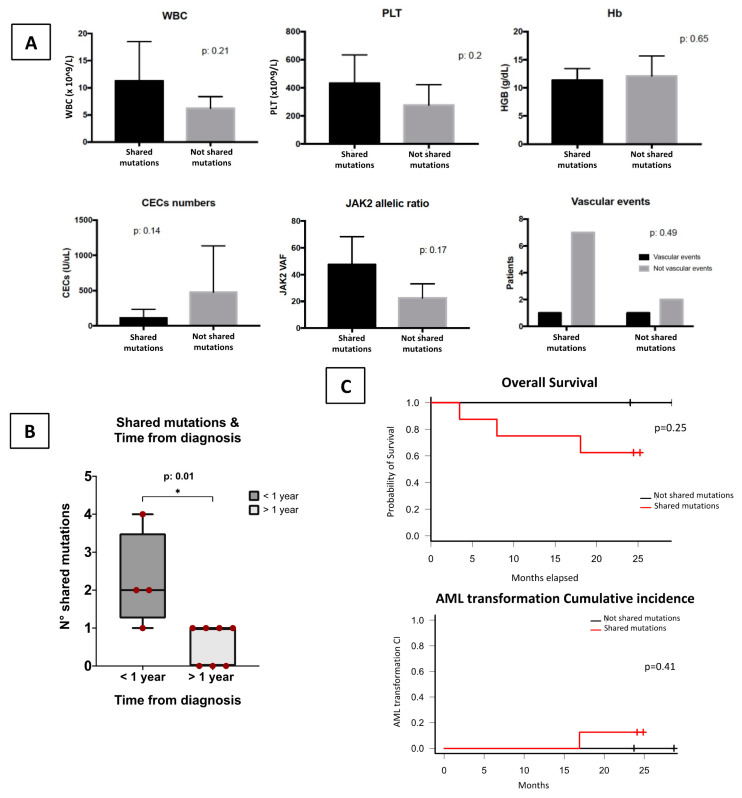
CECs Molecular profile and Clinical correlations. (**A**) No significative clinical or biological differences at baseline were found between patients who shared mutations between HSPCs and CECs and those who did not. (**B**) Number of shared mutations between CECs and HSPCs, according to the time from diagnosis. Patients collected within 1 year from PMF diagnosis shared an higher number of mutations between the two subpopulations compared with patients collected after 1 year (*p* = 0.01) (**C**) The presence of shared mutations not impact in clinical outcome of the PMF patients during the follow up (neither overall survival or Acute myeloid transformation cumulative incidence). Notably, all the patients who did not share any mutations between HSPCs and CECs are all still alive at the time of the analysis. WBC = White blood count; PLT = Platelets; Hb = Hemoglobin; CEC = Circulating endothelial cells; VAF = variant allele frequency; AML = Acute myeloid leukemia. * *p* < 0.05.

At baseline, no clinical differences were found between patients who shared mutations in HSPCs and CECs and those who did not (Figure 5A). Moreover, the presence of the *JAK2 V617F* on CECs was not related to any particular clinical or laboratory characteristics.

Notably, patients with the samples collected within 1 year from PMF diagnosis presented a higher number of shared mutations (*p* = 0.01) (Figure 5B). In particular, the patients who shared the highest number of mutated genes (included *JAK2*) were studied within 4 months from diagnosis, while the patients who didn’t share any mutations between CECs and HSPCs were collected at 26, 35 and 211 months (Supplementary Table S2).

The presence of shared mutations between CECs and HSPCs did not apparently impact on outcome, neither for the overall survival (*p* = 0.25) nor for the acute myeloid transformation cumulative incidence (Figure 5C). At 1 year from samples collection 75% of patients with shared mutation were alive [95%CI: 32–93], while no mortality was registered in patients who do not share any mutations. No vascular events were observed in all patients during the follow up. 

## 4. Discussion

Even though significant advances have been made in understanding the biology of PMF, the mechanisms underlying the high incidence of vascular events and the BM-spleen neoangiogenesis remain largely unexplained. Some authors have tried to answer these questions by looking at the *JAK2* MPN driver mutation in EPCs [16,17,18,23,24] or mature ECs captured by laser microdissection [21,25]. Overall, the results of these studies suggest an hypothetical direct ECs involvement in PMF pathogenesis [13,14]. However, difficulties in evaluating the “true” EPC or the limitations in studying “in vivo” mature ECs do not permit the clear demonstration of the endothelium implication in PMF. 

The aim of the MyCEC0617 study was to comparatively investigate the genomic profile of CD34+ enriched HSPCs and ECs in an attempt to trace a biological and possibly a pathogenetic link between these two cell populations in PMF. For the first time, the somatic mutational profile of the CECs isolated from PMF patients have been compared with the same one of paired HSPCs. Thanks to the high sensitivity and efficacy of CellSearch system in detecting CECs (CECs were detected in all samples) and of DEPArray system in sorting them (84.2% successful rate) we were able to overcome the limit and the ethical concerns of using laser microdissection for studying mature ECs, and to develop a new methodological approach for evaluating the mutational genome profile of these two different cell populations. 

The CellSearch technology combines the two traditional methods used to isolate CECs (i.e., anti CD146-immunomagnetic and immunofluorescent selection) and it’s the only single cell detection method approved by Food and Drug Administration [43]. Being a semi-automated system, it guarantees standardization in CECs identification and high-level of reproducibility, specificity and sensitivity [27,34]. Moreover, previous gene expression profiling (GEP) studies already validated the true endothelial origin of CECs isolated by CellSearch [44]. 

In the PMF patients, significant higher levels of CECs (25.5/mL), compared with healthy controls (4.25/mL) [*p* = 0.001] were detected. This result is consistent with previous findings [27], suggesting an endothelium damage in PMF [45]. In addition, a trend between a previous history of vascular events and CECs levels was also observed, although there was no significant difference. Previously, some other authors report an higher levels of CECs in patients with cardiovascular disease [46], reinforcing the role of CECs as markers of endothelial damage. 

Turning to the CECs molecular analysis, the first significant result of our study was that only the CECs from PMF patients presented MPN-related genes mutations, while no genomic alterations were found in the CECs isolated from the healthy controls. These findings strongly suggest that the acquisition of myeloid-associated genes mutations is strictly related to the PMF development. 

Notably, considering all the CECs analyzed, 28 different genes of the 54 genes panel were found to be mutated in PMF patients (sometimes the same mutation was found in several patients, i.e., TET2 in 4 patients; Figure 3B). This number was similar to the one observed in paired HSPCs (24 of 54 genes were mutated, Figure 3A). Moreover, PMF patients shared several myeloid-associated mutations between CECs and HSPCs. 

Considering the MPN driver mutations, 2 of the 6 *JAK2*^+^ patients (33.3%) shared the *JAK2 V617F* between HSPCs and CECs, while neither *MPL* nor *CALR* mutations were detected in the CECs. Notably, the patients with *JAK2* positive HSPCs/CECs were studied after few months from diagnosis and had also the higher number of mutated genes (9 and 8) and the higher number of shared mutations (4 and 3, respectively). The *JAK2 V617F* mutation was previously described in mature ECs in patients with MPNs [21,25]. In particular, the patients analyzed by Rosti [21] showed at least one EC harboring the *JAK2* mutation, but not all the ECs analyzed carried out it, suggesting that the endothelium of MPN patients may be composed by a mix of wild-type and *JAK2* mutated ECs. Considering the CECs, they derive from the whole body vessels, thus from both tissue involved and not by the disease. Therefore, the mutated ECs may represent a very low fraction of CECs, making difficult to identify the mutations with NGS. All these aspects may explain why we did not observe the *JAK2* driver mutation in the CECs of all patients and why we did not find a clear correlation with a previous history of thrombosis and /or splenomegaly. Our findings are in line with the observations of Sozer [25] and Rosti [21], while differ from Teofili’s study, in which the *JAK2* positive ECFCs were described only in a subset of patients with thrombosis [23]. 

Considering the non-driver MPN somatic mutations in the CECs, *ASXL1, TET2* and *SRSF2* genes were among the most frequently shared mutations and are also known to be the most frequently mutated genes in Myelofibrosis [3]. 

Notably, patients with samples collected within 1 year from PMF diagnosis presented an higher number of shared mutations (*p* = 0.01). These results may suggest that during the disease progression, the PMF clones and the EC clones might independently be lost or acquire growth advantages/disadvantages over time. At the same time, it may also be possible that patients not sharing somatic mutations on CECs and HSPCs may have a more indolent course resulting in a longer survival, while patients harboring shared mutations may have an adverse outcome early in the disease course. Additional prospective, systematic and larger studies will be needed to better clarify this aspect. Finally, the study of polymorphic alleles showed that LOH is a rare phenomenon in the studied setting of PMF patients and it affects only CECs. HSPCs did not present LOH. However, the low number of patients and the limits deriving from the study of only few loci did not allow any speculation on this data. Even though the clinical impact of somatic mutations on CECs or HSPCs was not among the objectives of our study, we analyzed the role of shared and un-shared somatic mutations on CECs in our cohort of patients and we did not find any relationship between the patients clinical and biological characteristics, vascular events, disease progression or survival and the number or the type of mutated genes in the HSPCs and CECs. 

Considering the HSPCs, their molecular profile was in line with the ones described in literature for PMF patients [3]. The absence of *CALR* on HSPCs analyzed may derive from the know technical difficulties on detecting this mutation with NGS [47,48]. Notably, all the healthy controls presented only known polymorphisms on HSPCs. 

Altogether, the presence of myeloid-associated mutations only in CECs from PMF patients, the frequency of mutated genes in CECs, similar to the ones described in PMF [3], and the high frequency of patients who shared at least one mutation between HSPCs and CECs, support a primary involvement of ECs in PMF. However, how the ECs may acquire myeloid-associated gene mutations remain an open question. An intriguing hypothesis already proposed in previous studies is that HSPC and ECs may originate from a common precursor cell, known as the “hemangioblast” [49]. However, its existence is still debated [50,51]. The detection of *JAK2 V617F* in ECs or EPCs from MPN patients may support this theory. Moreover, the recent evidence that *JAK2* mutation was acquired in utero or childhood in MPN patients [52,53] may be at least chronologically consistent with involvement of “hemangioblast” by MPN driver mutations. We think that our data give new significant elements supporting the Murray’s hypothesis. Indeed, (1) the high frequency of patients who shared at least one mutation between CECs and HSPCs (73%), (2) the number of mutations shared per patients (up to 4/patient) and the (3) presence of myeloid-associated mutations on CECs strongly support the hypothesis of a common precursors between HSPCs and ECs, which might act as the cell of origin of PMF. 

It has to be said that other mechanisms might explain the detection of myeloid associated mutations in ECs. One of them refers to the ability of monocytes of generating cells that closely resemble ECs, the so called “endothelial like cells” (ELCs) or angiogenic monocytes [54]. However, in humans it is currently thought that ELCs influence angiogenesis by secreting pro-angiogenic factors, rather than directly participate in neovascularization [55]. Moreover, the high frequency of shared mutations in our cohort and the presence also of different mutations between the two cell subpopulations, make this hypothesis unlikely. Other possible mechanisms might be the fusion of mutated hematopoietic cell with an EC or the phagocytosis of cell-free DNA or extracellular vesicles [56,57], but they also seem very unlikely, considering the complexity and variability of the CECs molecular profile. 

Regardless of the existence or not of a common precursor, the presence of somatic mutations in ECs may have important consequences in the disease development and the insurgence of vascular complications in PMF patients. Indeed, mutated ECs in PMF may represent a “neoplastic” vascular niche, which allow blood cells adhesion, vascular complications and the tumor cell growth, as demonstrated for *JAK2* -mutated ECs using in vitro and in vivo assays [14,58,59,60,61,62]. A longer follow up of our patients and new studies investigating the “neoplastic” vascular niche in humans are needed to validate this hypothesis. 

The small number of CECs collected in some patients and the low sensitivity of NGS are the main limitations to clearly say whether some mutations found in HSPCs and not in CECs, or vice versa, are the result of mutational heterogeneity. Probably, only a part of the CECs collected derive from mutated EC involved with the disease and also this factor could make difficult to analyze the molecular profile of the CECs and compare it with the one of HSPCs. 

However, on the other hand, we think that the discovery of shared and un-shared somatic mutations, despite the low number of CECs collected and the low NGS sensitivity, highlights the ECs involvement in MF and reinforce the hypothesis of a common precursor between ECs and HSPCs. Increasing the number of analyses, it cannot be excluded that this involvement may be even higher and that the mutations shared between CECs and HSPCs may be more. Thus, new and larger studies specifically aimed to evaluate the frequency of HSPCs and CECs shared mutations and its correlation with clinical characteristics of disease are needed.

In conclusion, our study through a new methodological approach describes for the first time the genomic mutational profile of both HSPCs and CECs in PMF patients and provides new knowledge on the cell of origin in myeloproliferative neoplasms and the potential role of ECs in the “neoplastic” vascular niche. These preliminary results have also a particular value because they open to further studies aiming to clarify the clinical relevance of the reported mutational status in the two populations and provide new insights into the mechanisms for the shared mutations. In doing so, it will be necessary to expand the cases and create an animal model for functional studies. 

## Figures and Tables

**Table 1:** Patients and healthy controls characteristics.

Features	PMF Patients	Healthy Controls	*p Value*
	N or Median *(% or Range)*	N or Median *(% or Range)*	
Age (years)	71.5 *(54–85)*	65 *(35–84)*	*0.22*
Male	9/14 *(64%)*	1/5 *(20%)*	*0.14*
PMF	14/ 14	0/5	
Months from Diagnosis	20.5 *(1–211)*	*NA*	
WBC (×10^9^/L)	7.3 *(3.8–117)*	5.5 *(3.9–9.1)*	*0.35*
Hb (g/dL)	10.7 *(8–14.8)*	13.6 *(12–14.5)*	*0.01*
PLT (×10^9^/L)	211 *(50–885)*	257 *(179–412)*	*0.77*
Constitutional Symptoms	4 *(29%)*	*NA*	
Altered karyotypes	3 (*21%*)	*NA*	
Previous Thrombosis	2 *(14%)*	*0 (0%)*	*0.99*
Splenomegaly			
*N° patients*	11 *(79%)*	0 *(0%)*	
*cm below LMC*	5 *(0–16)*	0	
Treatment			
*Hydroxyurea*	4 *(29%)*	0 *(0%)*	
*None*	10 *(71%)*	5 *(100%)*	
BM fibrosis			
*WHO grade 1*	7 *(50%)*	*NA*	
*WHO grade 2*	6 *(43%)*	*NA*	
*WHO grade 3*	1 *(7%)*	*NA*	
DIPSS (at samples collection)			
*Low*	0 *(0%)*	*NA*	
*Intermediate 1*	7 *(50%)*	*NA*	
*Intermediate 2*	5 *(36%)*	*NA*	
*High*	2 *(14%)*	*NA*	
Driver Mutations			
*JAK2*	9 *(64%)*	*NA*	
*CALR*	2 *(14%)*	*NA*	
*MPL*	2 *(14%)*	*NA*	
*Triple negative*	1 *(7%)*	*NA*	

PMF Patients and healthy controls characteristics; PMF = Primary Myelofibrosis; BM = bone marrow; WBC = White blood count; Hb = Hemoglobin; PLT = Platelets.

**Table 2:** Impact of the patients’ characteristics on the CECs detection.

Features	PMF Patients		Healthy Controls		*p Value*
	CEC Median (*Range*); n pts	*p Value*	CEC Median (*Range*); n pts	*p Value*	
CECs detected	109 (15–1448); *n* = 14		17 (11–19); *n* = 5		*0.001*
CECs collected	16.5 (0–118); *n* = 14		8 (2–11); *n* = 5		*0.6*
Sex		*0.53*		*NA*	
Male	120 (31–1448); *n* = 9		17; *n* = 1		*NA*
Female	116 (54–290); *n* = 5		16 (11–19); *n* = 4		*0.02*
Age		*0.21*		0.2	
≥70 years	54 (15–399); *n* = 7		12 (11–13); *n* = 2		*0.06*
<70 years	120 (22–1448); *n* = 7		19 (17–19); *n* = 3		*0.02*
Time from diagnosis		*0.62*			
<2 years	67 (21–399); *n* = 7		*NA*		
>2 years	116 (15–1448); *n* = 7		*NA*		
White blood count		*0.36*		*NA*	
>10 × 10^9^/L	67 (11–1448); *n* = 5		0		*NA*
≤10 × 10^9^/L	123 (15–1448); *n* = 9		17 (11–19); *n* = 5		*0.007*
Constitutional symptoms		*0.95*			
Yes	93.5 (22–399); *n* = 4		*NA*		
No	109 (15–1448); *n* = 10		*NA*		
History of thrombosis		*0.30*			
Yes	217.5 (21–399); *n* = 4		0		
No	84.5 (15–1448); *n* = 10		17 (11–19); *n* = 5		
Splenomegaly		*0.99*			
Yes	116 (15–1448); *n* = 11		0		
No	102 (22–290); *n* = 3		17 (11–19); *n* = 5		
Treatment		*0.94*			
Hydroxyurea	102 (54–290); *n* = 5		0		
No treatment	116 (15–1448); *n* = 9		17 (11–19); *n* = 5		
DIPSS		*0.90*			
Interm1	116 (25–145); *n* = 7		*NA*		
Interm2-High	102 (21–1448); *n* = 7		*NA*		
Driver mutations		*0.30*			
JAK2	67 (15–399); *n* = 9		*NA*		
Non JAK2 mutations	120 (22–1448); *n* = 5		*NA*		

The mean of CECs isolated was in 4 mL of peripheral blood ± SEM. The thresholds have been chosen as follow: for the age it was based on the median age of the entire cohort (71 years), while for the WBC it was based on the upper limit of normality of our laboratory (10 × 10^9^/L). The threshold for the time from diagnosis is 2 years because the median time from diagnosis to sample collections was 26 months. SEM = standard error of the mean; *n* = number; pts = patients; HCs = healthy controls; Interm = intermediate. The analysis was performed using the Mann-Whitney test.

## Data Availability

For original data, please contact mirkfar@gmail.com.

## Supplementary Materials

The following are available online at https://www.mdpi.com/article/10.3390/cells10102764/s1, Table S1: Patients and controls characteristics at the time of samples collection; Table S2: Patients’ characteristics and mutations detected on CECs and HSPCs.

## Appendix A. Circulating Endothelial Cell Identification by CellSearch Protocol

The CellSearch system provides the following step s [34].

10 mL of peripheral blood is drawn into a specific CellSearch conical tube and shipped overnight to a central Laboratory (Menarini Laboratory, Bologna, Italy). The CellSearch system consists of two instruments: the CellTrack Autoprep and the Analyzer.

At the central laboratory, 5.5 mL of CellSearch dilution buffer are added to the peripheral blood and centrifuged at 800× *g* for 10 min without brake. Thereafter, the tube is carefully loaded into the AutoPrep system and the diluted plasma will be removed until 1 cm above the red blood cell layer. Then, anti-CD146 ferrofluid and dilution buffer are added to the tubes and mixed by pipetting. The ferro-fluid reagent consists of nanoparticles with a magnetic core surrounded by a polymer layer coated with antibody directed towards the CD146 antigen for the selection of the CECs. CD146, also known as the melanoma cell adhesion molecule (MCAM), is a cell adhesion molecule currently used as a marker for endothelial cell lineage. Then, the magnets are moved back and forward towards the tube to enhance the collisions between cells and ferrofluids. After an incubation period, the magnets remain against the tube, anti-CD146-ferrofluids and the cells that have bound ferrofluid will be pulled to the magnets, and the rest of the cells are removed in a single pipetting step. Thereafter, the enriched cells were fluorescently labelled with the nuclear stain 4,6-diamidino-2- phenylindole (DAPI). The others immunofluorescent reagents were anti-CD105-PE, which is specific for the protein endoglin that is expressed by activated endothelial cells, activated monocytes, stromal cells and pre-B cells, and anti-CD45-APC, to identify leucocyte. Therefore, staining reagents (<0.0006% mouse monoclonal antibodies specific to CD105 conjugated to phycoerythrin; <0.0013% mouse antiCD45 monoclonal antibodies conjugated to allophycocyanin in phosphate-buffered saline containing 0.5% BSA and 0.1% sodium azide) are added in conjunction with a permeabilization buffer to label the cells fluorescently. After incubation, magnetic separation is repeated to remove the excess staining reagent. After the final processing step, the cells are re-suspended in 300 uL of buffer and transferred to a chamber placed between two magnets that orientate the immunomagnetically labelled cells in a monolayer for analyses. The cells are then examined with a four-color semi-automated fluorescent microscope, the CellSpotter Analyzer II. A grey-scale charge-coupled device camera is used to scan the entire chamber surface, and each captured frame is then evaluated for potential CEC candidates by image analysis software (Figure 2D). In summary, CECs were defined as CD146+DAPI+CD105+CD45- cells. On the contrary, leukocytes were described as CD146+DAPI+CD105-CD45- cells.

## Appendix B. CECs Collection with DEPArray System Protocol

The DEPArray System (Di-Electro-Phoretic Array system; by Menarini Silicon Biosystems) [35] can analyze samples containing from one to tens of thousands of cells and the DEPArray analysis platform utilizes high quality, image-based selection to identify and isolate the cells of interest. In detail, the DEPArray System is composed of three elements: a benchtop instrument, a disposable microfluidic cartridge and a proprietary software, the CellBrowser. The working principle of the DEPArray is the Dielectrophoresis (DEP), an electrokinetic principle based on the ability of a non- uniform electric field to exert forces on neutral, polarizable particles, such as cells, which are suspended in a liquid. The core of the technology is the microsystem cartridge, which is a single-use device integrating a microelectronic silicon chip, microfluidic chambers and valves. The silicon substrate in the cartridge integrates an array of over 300,000 micro-electrodes, each electrode can be programmed and energized with Alternating Current in-phase or counter-phase voltages with respect to the glass lid, which is conductive and transparent. By applying an appropriate pattern of phases, the array can generate up to 30,000 “DEP cages” in the Main Chamber, each one able to capture a cell in stable levitation, avoiding contacts between the cells and surfaces during the sorting process. DEP cages are able to trap and move cells of different type and size ranging from small sperm cells to large epithelial cells [63,64,65]. This electronic structure is integrated within an innovative microfluidic architecture that includes three micro-chambers in fluidic connection: the Main Chamber (where the sample is loaded), the Parking Chamber (where the target cells are collected before the recovery) and the Recovery Chamber.

Briefly, to allow loading of samples from CellSearch cartridges in a DEPArray cartridge, CellSearch CEC samples were aspirated from their CellSearch cartridge using a 200 mL gel loading tip pre-rinsed in a 2% BSA in PBS solution. The whole suspension was centrifuged for 10 min at 300 g, cells were washed once in 1 mL of SB115 buffer (a proprietary low-conductivity buffer for sorting fixed cells in the DEPArray cartridge) and finally re-suspended in 14 mL of SB115 buffer. Thereafter, DEPArray cartridges were manually loaded with 14 mL of sample and 800 mL of the buffer solution in which purified or single cells had to be recovered. After loading the cartridge into the DEPArray system, 9.26 mL of sample was automatically injected by the system into a microchamber of the cartridge where the cells were spontaneously organized into a preprogrammed electric field consisting of 16,000 electrical cages in which individual cells are trapped. Image frames covering the entire surface area of the microchamber for each of three fluorescent filter cubes (PE, APC and DAPI/Hoechst) and bright field images were captured. Cells were automatically detected by the system based on a DAPI/Hoechst fluorescence threshold and were assigned a unique cell ID. Captured images were digitally processed and presented in a software module that enables selection of cells of interest by the operator. Next, for recovery selected cells were moved simultaneously to a parking area adjacent to the main microchamber in the cartridge. Individual cells or groups of cells were subsequently moved to a recovery area where a last visual confirmation of cell presence can be performed. To recover group of cells, the content of the recovery area was flushed with two drops of buffer (ca. 30–40 mL) into a 200 mL PCR tube. The entire cell routing process was monitored under bright field imaging.

The proprietary CellBrowser software enables an automatic or operator-assisted identification of the desired cells through the elaboration of high-resolution images, minimizing the possibility to select inappropriate events, such as debris and doublets. The different cell populations are selected by using a manual or semi-automatic gating. Once identified, each target cell can be isolated from the bulk population, automatically, in the following way: the instrument moves the selected DEP cages (containing the target cells) by changing the electric field pattern step by step, deterministically, concurrently and independently along trajectories calculated by the software, moving each selected cell from the original location into the Parking chamber. Afterwards, cells can be displaced, as single-cells or in pools of up to 507 cells. At the end of the process, the target cells can be eluted from the device directly into various types of supports, through an accurate microfluidic control, by flowing clean buffer loaded in the cartridge prior to use. The recovery procedure can be repeated to obtain from the same sample multiple separate recoveries of individual target cells (up to 96) and/or groups of cells [1]. In contrast with other traditional bulk sorting, DEPArray™ technology isolates single and pure cell populations. The high-quality and accuracy of DEPArray™ technology has been thoroughly validated by using immunofluorescence and molecular- based approaches, with both spike in and real biological samples [63].

## Appendix C. Protocol for DNA Extraction, Amplification and NGS Analysis

DNA extracted from isolated CEC and HSPC was then amplified in order to obtain a quantity suitable for NGS analysis. The Whole Genome Amplification (WGA) was performed by Reply-g DNA library kit (Qiagen) following “Amplification of Genomic DNA from Single Cells” procedure. Our approach was based on the gene target capture sequencing. Specific probes (NimbleGen by Roche) have been used in order to hybridize all exons of the above-mentioned genes (141 kb).

Briefly, up to 1000 cells were resuspended in PBS and treated by denaturating solution, which allow the membrane degradation and the DNA denaturation. This phase was followed by WGA obtained using Phi29 TaqPolymerase380. The WGA will take 3 h and may be concluded with tagmentation, end-repair and A-tailing procedures in order to produce NGS library or stopped. Amplified genomic DNA is stable and NGS analysis may be subsequently performed.

DNA was first analyzed by MiSeq Illumina NGS platform, specific and sensitive to study multiple target genes when low amount of DNA is available. Firstly, 300 ng of amplified genomic DNA from CECs or HSPCs was screened for mutations in 54 genes known to be associated to Myelofibrosis [3,4,31,66,67,68] (Figure 1B). DNA was tagmented by enzimatic reaction. The fragmentation was immediately followed by end-repair reaction and the index and adaptors ligation. Index and adaptors are small sequences of DNA that need to be associated to the amplicon samples in order to uniquely identify each sample during the sequencing and the data analysis and to be recognized by the software as “true read”. The DNA was then incubated with NimbleGen probes. The incubation was followed by the enrichment of the captured fragments, purifications by Ampure Beads and a final amplification. The captured sequences of CEC and HSPC DNA from 4 patients were thus pooled (8 samples per pool) [38] and sequenced following manufacturer’s instructions by MiSeq Illumina NGS platform using 2 × 150 sequencing (V2 kit, TruSeq). One sequencing run was required in order to sequence 8 samples with a coverage about 3200× [39]. The .vcf files were analyzed using the free bioinformatics tool wAnnovar (Wang Genomics Lab 2010–2020) [40,69]. The cutoffs to confirm the presence of the mutations were identification of mutant alleles in 30 and 50 reads both in forward and reverse, for HSPCs and CECs, respectively.
