# Supplementary material for: Comparative Mutational Profiling of Hematopoietic Progenitor Cells and Circulating Endothelial Cells (CECs) in Patients with Primary Myelofibrosis
Source: Cells. 2021 Oct 15;10(10):2764. doi: 10.3390/cells10102764 (PMC8534986; doi:10.3390/cells10102764)
Supplement: Supplementary file 1 [file cells-10-02764-s001.zip › cells-1339249-supplementary.pdf]

Supplemental Materials

Supplemental Table S1

Patients and controls characteristics at the time of samples collection

For inclusion in the MyCEC0617 study, patients and healthy controls must fulfill the following criteria: (a) subjects must be over 18 years old, (b) have a performance status greater or equal to 2 (ECOG score), (c) and patients being diagnosed with primary myelofibrosis, (d) but not being treated with JAK2 inhibitors (treatment with Hydroxyurea was permitted). The inclusion criteria were thought to avoid possible bias or confounding factors deriving by the use of JAK2 inhibitors or by previous myeloproliferative disease (i.e., Polycythemia Vera or Essential Thrombocythemia).

All patients were diagnosed with primary myelofibrosis. Half of the patients presented also other comorbidities. In detail, 2 presented an atrial fibrillation, while 2 with metabolic syndrome, one had a low left ventricular function, one had chronic obstructive pulmonary disease, one with type II diabetes mellitus and one had Basedow disease. Most of patients had a low or intermediate grade of bone marrow fibrosis. Indeed, 13 patients presented a grade 1 or 2 of bone marrow fibrosis, according to the World Health Organization classification.

More than 70% of patients did not receive any treatment at the time of samples collection, while four patients were receiving hydroxyurea, as cytoreductive treatment. In median, they were under treatment for 12 months (6-24). The treatment was well tolerated and no side effects were referred. No statistical differences were found between patients and controls considering Age (p: 0.24), Sex (p: 0.14), Previous history of thrombosis (p: 0.99) or blood count (p: 0.35).

PMF=Primary Myelofibrosis; HC=healthy control; M=male, F=Female; Intm=intermediate; Y=Yes; N=No; HU=Hydrossiurea; NA=Not applicable; BM=bone marrow; WBC=White blood count; Hb=Hemoglobin; PLT=Platelets

Supplemental Table S2

Patients' characteristics and mutations detected on CECs and HSPCs with their respective Variant allele frequency (VAF). The mutations shared between CECs and HSPCs are in bold.

Supplemental Table S1

	MYCEC_01	MYCEC_02	MYCEC_03	MYCEC_04	MYCEC_05	MYCEC_06	MYCEC_07	MYCEC_08	MYCEC_09	MYCEC_10	MYCEC_11	MYCEC_12	MYCEC_13	MYCEC_14	MYCEC_15	MYCEC_16	MYCEC_17	MYCEC_18	MYCEC_19
**Disease**	PMF	PMF	PMF	PMF	PMF	PMF	PMF	PMF	PMF	PMF	PMF	PMF	PMF	PMF	HC	HC	HC	HC	HC
**Months from diagnosis**	1	2	4	4	15	28	28	31	211	28	35	1	26	1	NA	NA	NA	NA	NA
**Driver Mutation**	JAK2	JAK2	JAK2	JAK2	JAK2	JAK2	JAK2	JAK2	JAK2	CALR	CALR	MPL	MPL	Triple neg	NA	NA	NA	NA	NA
**JAK2 allelic ratio**	27,3	55%	33%	77%	63%	35%	22%	76%	30%	NA	NA	NA	NA	NA	NA	NA	NA	NA	NA
**Sex**	F	M	M	F	F	M	M	M	M	F	M	M	F	M	F	M	F	F	F
**Age** (years)	81	78	72	64	85	57	74	78	72	61	66	54	71	64	35	46	65	77	84
**DIPSS risk class**	Intm-2	Intm-2	Intm-2	Intm-1	Intm-1	High	Intm-1	Intm-1	Intm-1	Intm-1	Intm-2	Intm-2	Intm-1	High	NA	NA	NA	NA	NA
**Splenomegaly**	Yes	Yes	Yes	Yes	Yes	No	Yes	Yes	Yes	Yes	Yes	Yes	No	No	No	No	No	No	No
**Spleen (cm below LCM)**	3 cm	5 cm	9 cm	6 cm	5 cm	0 cm	16 cm	3 cm	3 cm	10 cm	9 cm	10 cm	0 cm	0 cm	0 cm	0 cm	0 cm	0 cm	0 cm
**Constitutional symptoms**	No	Yes	Yes	No	No	No	No	No	No	No	No	Yes	No	Yes	NA	NA	NA	NA	NA
**Previous thrombosis**	No	No	Yes	No	No	No	No	No	Yes	No	No	No	No	No	No	No	No	No	No
**Treatment**	None	HU	None	None	HU	HU	None	None	HU	None	None	None	None	None	None	None	None	None	None
**BM fibrosis**	1	1	2	2	2	1	2	1	1	2	3	1	1	2	NA	NA	NA	NA	NA
**WBC** (x10^9/L)	5,6	19,5	3,8	6,27	11	10,9	4,7	7,5	7,3	5,9	6,8	60	7,6	117,1	5,4	5,6	6,6	3,9	9.1
**Hb** (g/dL)	8,1	12,3	8,5	12,9	13	9,1	10,7	10,9	14,8	10,6	8	9,8	12,4	8,9	13	14,5	13,6	14,4	12
**PLT** (x10^9/L)	210	144	266	394	855	101	201	234	183	624	163	440	707	50	370	225	257	179	412

Supplemental Table S2

Patients	Condition	Driver Mutation	Time from diagnosis *(months)*	Spleen *(cm under LCM)*	Previous thrombosis	CD34+ HPSCs (nx10^4/ml)	CECs detected (n/4ml)	CECs recovered (n/4ml)	Mutation on CD34+ HPSC *name (mutation; VAF%)*	Mutation on CEC *name (mutation; VAF%)*
MYCEC_01	PMF	JAK2	1	3 cm	No	6.7	21	0	- - - -	- - - -
MYCEC_02	PMF	JAK2	2	5 cm	No	6	67	29	***MPN driver mutations shared*** **:**<br>**JAK2 (c.G1849T:p.; 61%)**<br>***NON MPN driver mutations shared*** **:**<br>**IDH1(c.A593G:p.; 46.4%)**<br>**ABL1 (c.T2035G:p.; 45.9%)**<br>**TET2 (c.C3781T:p.; 38.2%)**<br>*Somatic mutations* :<br>TET2 (c.822delC:p.; 39.9%)<br>ASXL1(c.1927dupG:p.; 34%)<br>SRSF2 (c.C283G:p.; 38.2%)	***MPN driver mutations shared*** **:**<br>**JAK2 (c.G1849T:p.; 16%)**<br>***NON MPN driver mutations shared*** **:**<br>**IDH1(c.A593G:p.; 19%)**<br>**ABL1(c.T2035G:p.; 5%)**<br>**TET2 (c.C3781T:p.; 32%)**<br>*Somatic mutations* :<br>CSF3R(c.1404dupC:p.;11%)<br>CBLB (c.G1613A:p.; 17%)<br>KMT2A (c.2312dupC:p.; 13%)<br>SRSF2 (c.287dupC:p.; 17%)<br>SETBP1 (c.645dupC:p.;20%)
MYCEC_03	PMF	JAK2	4	9 cm	Yes	7.2	399	118	***MPN driver mutations shared*** **:**<br>**JAK2 (c.G1849T:p.; 28%)**<br>***NON MPN driver mutations shared*** **:**<br>**ASXL1 (c.C1249T:p.; 26%)**<br>**KMT2A (c.C89G:p.; 26%).**<br>*Somatic mutations* :<br>NOTCH1 (c.G6396C:p.; 52%)<br>CEBPA(c.232_233insACCCGC:p.; 45%)<br>ATXR (c.T4997A:p.; 18%)	***MPN driver mutations shared*** **:**<br>**JAK2 (c.G1849T:p.; 97%)**<br>***NON MPN driver mutations shared*** **:**<br>**ASXL1 (c.C1249T:p.; 47%)**<br>**KMTA2 (c.C89G:p.; 20%)**<br>*Somatic mutations* :<br>JAK3(c.G3285T:p.; 62%; c.C2677G:p.; 21%; c.G1611A:p.; 25%)<br>TET2 (c.5254delA:p.; 11%)<br>CUX1 (c.1316dupC:p.; 12%)<br>KMTA2 (c.2312dupC:p.; 30%)<br>FLT3 (c.G1484A:p.; 35%)<br>STAG2 (c.C1802T:p.; 46%)
MYCEC_04	PMF	JAK2	15	6 cm	No	8.2	123	25	*MPN driver mutation* : None<br>***NON MPN-driver mutations shared*** **:**<br>**WT1 (c.G362A:p.; 48%)**<br>*Somatic mutations* :<br>PDGRFA (c.C2778T:p.; 55%)<br>NOTCH1 (c.C1023G:p.; 21%)<br>CBLB (c.G1865C:p.; 52%)	*MPN driver mutation* : None<br>***NON MPN-driver mutations shared*** **:**<br>**WT1 (c.G362A:p.; 14%)**<br>*Somatic mutations* :<br>U2AF1 (c.G461A:p.; 23%)<br>KDM6A (c.G2056A:p.; 81%)<br>SMC1A (c.G2820A:p.; 25%)<br>ATRX (c.C3817A:p.; 19%)
MYCEC_05	PMF	JAK2	28	5 cm	No	12.7	54	8	*MPN driver mutation* :<br>JAK2 (c.G1849T:p.; 40%)<br>***NON MPN-driver mutations shared*** **:**<br>**KIT (c.T2805A:p.; 45%)**<br>*Somatic mutations* :<br>TET2 (c.C3100T:p.; 41%)<br>SF3B1 (c.T1155G:p.; 55%)<br>ASXL1 (c.1927dupG:p.; 39%)<br>BCORL (c.A1111C:p.28%)	*MPN driver mutation* :<br>None<br>***NON MPN-driver mutations shared*** **:**<br>**KIT (c.T2805A:p.; 75%)**<br>*Somatic mutations* :<br>ATXR (c.3031dupA:p.; 33%)
MYCEC_06	PMF	JAK2	4	0 cm	No	0.7	102	5	*MPN driver mutation NOT shared* :<br>JAK2 (c.G1849T:p.; 28%)<br>***NON MPN driver mutations shared*** **:**<br>**SRSF2 (c.C284T:p.; 37.5%)**<br>*Somatic mutations* :<br>NOTCH1 (c.A311G:p.; 42%)<br>ASXL1 (c.A2957G:p.; 53%)<br>RUNX1 (c.C1193T:p.; 26%)	*MPN driver mutation* :<br>None<br>***NON MPN driver mutations shared*** **:**<br>**SRSF2 (c.C284T:p.; 21%)**<br>*Somatic mutations* :<br>JAK2 (c.1498dupC:p.; 20%)<br>RAD21 (c.G1749C:p.; 21%)<br>TP53 (c.G218A:p.; 59.5%)
MYCEC_07	PMF	JAK2	31	16 cm	No	9.8	15	4	*MPN driver mutation* :<br>JAK2 (c.G1849T:p.; 17%)<br>***NON MPN-driver mutations shared*** **:**<br>**TP53 (c.G131A:p.; 12%)**	*MPN driver mutation* :<br>None<br>***NON MPN-driver mutations shared*** **:**<br>**TP53 (c.G131A:p. 28%)**<br>*Somatic mutations* :<br>ASXL1 (c.C369A:p.; 42%).<br>KDM6A (c.G3409A:p.; 83%)
MYCEC_08	PMF	JAK2	28	3 cm	No	0.9	31	0	- - - -	- - - -
MYCEC_09	PMF	JAK2	211	3 cm	Yes	5.7	145	27	*MPN driver mutation* :<br>JAK2 (c.G1849T:p.; 30.7%)<br>*Somatic mutations* :<br>ATAD3A(c.C1238T:p.; 5%)<br>PDGRFA (c.G1731A:p.; 48%)<br>FLT3 (c.T11A:p.; 27%)	*MPN driver mutation* :<br>None<br>*Somatic mutations* :<br>MPL (c.C812A:p.; 30%)<br>SF3B1 (c.3066dupC:p.; 28.8%; c.2422dupA:p.; 37.6%)
MYCEC_10	PMF	CALR	28	10 cm	No	7.2	116	30	*MPN driver mutation* :<br>None<br>***NON MPN-driver mutations shared*** **:**<br>**NOTCH1 (c.C4197T:p; 47%)**	*MPN driver mutation* :<br>None<br>***NON MPN-driver mutations shared*** **:**<br>**NOTCH1 (c.C4197T:p; 60%)**<br>*Somatic mutations* :<br>IDH1 (c.G541A:p; 31%)<br>PTEN (c.C1076T:p; 7%)<br>PTPN11 (c.G214A:p; 17,5%)<br>SETBP1 (c.C3860T:p, 14%)<br>ASXL1 (c.1927dupG:p; 49%)<br>STAG2 (c.G2968A:p.; 8%)<br>JAK2 (c.T2267C:p.; 12%)
MYCEC_11	PMF	CALR	35	9 cm	No	6.3	1448	122	*MPN driver mutation* :<br>None<br>*Somatic mutations* :<br>FBXW7(c.A221C:p; 47%)<br>GNAS (c.C1268A:p; 33%)	*MPN driver mutation* :<br>None<br>*Somatic mutations* :<br>CSF3R (c.G2485T:p; 9%)<br>TET2 (c.1660dupC:p.; 24.4%; c.1842_1843insA:p.; 14.3%)<br>KMT2A (c.2312dupC:p.;16%)<br>TP53 (c.A182G:p.; 34%)
MYCEC_12	PMF	MPL	26	10 cm	No	1.6	120	0	- - - -	- - - -
MYCEC_13	PMF	MPL	1	0 cm	No	1.5	290	32	*MPN driver mutation* :<br>MPL (c.G1544T:p.; 14%)<br>*Somatic mutations* :<br>CSF3R (c.T2087C:p.; 49%)<br>ABL1^(c.C2429T:p.; 60%)<br>SRFS2 (c.C283G:p.; 19.5%)<br>ASXL1(c.1927dupG:p.;27.5%)	*MPN driver mutation* :<br>None<br>*Somatic mutations* :<br>TET2 (c.C5167T:p.; 33%)<br>KMT2A (c.C9673G:p.; 14%)<br>ASXL1 (c.C369A:p.; 17%)<br>ATRX (c.4630dupA:p.; 54%)<br>STAG2 (c.T231A:p.; 26%; c.G1126A:p., 79%)
MYCEC_14	PMF	triple neg	1	0 cm	No	3.1	22	1	*MPN driver mutation* :<br>None<br>***NON MPN-driver mutations shared*** **:**<br>**TET2 (:c.T2599C:p. 49%; c.C5167T:p. 55%)**<br>**NOTCH1 (c.C4930T:p 53%)**<br>*Somatic mutations* :<br>CALR (c.G566C:p; 53%)	*MPN driver mutation* **:**<br>None<br>***NON MPN-driver mutations shared*** **:**<br>**TET2 (:c.T2599C:p. 96%; c.C5167T:p. 97%).**<br>**NOTCH1 (c.C4930T:p; 96%)**
MYCEC_15	Normal control	NA	NA	0 cm	No	0.3	19	2	None	None
MYCEC_16	Normal control	NA	NA	0 cm	No	2.6	17	11	None	None
MYCEC_17	Normal control	NA	NA	0 cm	No	3.4	19	9	None	None
MYCEC_18	Normal control	NA	NA	0 cm	No	4.3	13	4	None	None
MYCEC_19	Normal control	NA	NA	0 cm	No	3.2	11	8	None	None
